# Individual participant data meta‐analysis of intervention studies with time‐to‐event outcomes: A review of the methodology and an applied example

**DOI:** 10.1002/jrsm.1384

**Published:** 2020-02-06

**Authors:** Valentijn M.T. de Jong, Karel G.M. Moons, Richard D. Riley, Catrin Tudur Smith, Anthony G. Marson, Marinus J.C. Eijkemans, Thomas P.A. Debray

**Affiliations:** ^1^ Julius Center for Health Sciences and Primary Care University Medical Center Utrecht, Utrecht University Utrecht the Netherlands; ^2^ Cochrane Netherlands, Julius Center for Health Sciences and Primary Care University Medical Center Utrecht, Utrecht University Utrecht the Netherlands; ^3^ Centre for Prognosis Research, Research Institute for Primary Care and Health Sciences, Keele University Staffordshire UK; ^4^ Department of Biostatistics University of Liverpool Liverpool UK; ^5^ Department of Molecular and Clinical Pharmacology University of Liverpool Liverpool UK

**Keywords:** heterogeneity, individual participant data, intervention, meta‐analysis, time‐to‐event

## Abstract

Many randomized trials evaluate an intervention effect on time‐to‐event outcomes. Individual participant data (IPD) from such trials can be obtained and combined in a so‐called IPD meta‐analysis (IPD‐MA), to summarize the overall intervention effect. We performed a narrative literature review to provide an overview of methods for conducting an IPD‐MA of randomized intervention studies with a time‐to‐event outcome. We focused on identifying good methodological practice for modeling frailty of trial participants across trials, modeling heterogeneity of intervention effects, choosing appropriate association measures, dealing with (trial differences in) censoring and follow‐up times, and addressing time‐varying intervention effects and effect modification (interactions).We discuss how to achieve this using parametric and semi‐parametric methods, and describe how to implement these in a one‐stage or two‐stage IPD‐MA framework. We recommend exploring heterogeneity of the effect(s) through interaction and non‐linear effects. Random effects should be applied to account for residual heterogeneity of the intervention effect. We provide further recommendations, many of which specific to IPD‐MA of time‐to‐event data from randomized trials examining an intervention effect.We illustrate several key methods in a real IPD‐MA, where IPD of 1225 participants from 5 randomized clinical trials were combined to compare the effects of Carbamazepine and Valproate on the incidence of epileptic seizures.

**Highlights**

**What is known?**

- Time‐to‐event (survival) data can be analyzed with Cox Proportional Hazards regression, but proportionality of hazards should be tested.
- Individual participant data (IPD) from multiple randomized trials can be summarized by meta‐analyzing the trial‐specific estimates of the individual trials (studies) or by analyzing the pooled data with a mixed‐effects model that accounts for between‐trial heterogeneity in intervention effect and frailty of participants.

**What is new?**

- We summarize published guidance, statistical methods and software for survival analysis using IPD from multiple randomized clinical trials.
- We discuss how between‐trial heterogeneity of intervention effects may appear and how its sources can be investigated.
- We illustrate the methods on real epilepsy data and provide R code.

**Potential impact for other fields**

- Meta‐analysis is not only relevant in medical research, but also in other research areas.
- The methods naturally extend to meta‐analysis of non‐randomized studies, where treatment effect estimates need to be adjusted for confounding.

## 1 INTRODUCTION

Relative intervention effects (eg, hazard ratios) are most reliably evaluated in randomized clinical trials (RCT). However, multiple RCTs of the same intervention may provide inconclusive or conflicting evidence on efficacy or safety. Discrepancies between evidence from different RCTs may arise due to chance, or in particular due to heterogeneity in the true intervention effect. This heterogeneity is commonly caused by across‐trial differences in, for example, study design (eg, recruitment strategy, length of follow‐up, or analysis methods), case‐mix of participants, definition of the studied outcome(s), the implementation (eg, dosage or intensity) of the intervention. This motivates the need to systematically integrate and summarize evidence across trials, to facilitate evidence‐based‐medicine.

This can be achieved using a systematic review with meta‐analysis (MA). Whereas most meta‐analyses are based on aggregated data (AD) from available literature, individual participant (or patient) data meta‐analyses (IPD‐MA) of multiple intervention studies are considered the gold standard. 1, 2, 3 IPD‐MA offers several advantages, as the meta‐analyst has full control of the data analysis and uses the data at the individual participant level. 4 Key advantages are the standardisation of outcome and follow‐up definitions, checking of data and quality, proper modelling of time‐to‐event outcomes, and the exploration of intervention‐covariate interactions at the participant level. 4, 5 It may thus come to no surprise that IPD‐MA are increasingly common. 6, 7

Extensive guidance has previously been provided for conducting an IPD‐MA of intervention effects, for various types of outcome data, such as binary, 7, 8, 9 continuous, 6, 7, 10, 11 ordinal 7 and count outcomes. 7 Yet, IPD‐MA are especially useful when analyzing time‐to‐event outcomes in intervention studies, as censored outcomes can be reassessed for the meta‐analysis, survival measures (eg, hazard ratios, median survival) can be calculated directly and independent to trial reporting, follow‐up length can often be increased, time‐varying hazard ratios can be examined, and effect modifiers (intervention‐covariate interactions) can be assessed. 12, 13

Whereas a wealth of methods have been developed for analyzing and predicting time‐to‐event outcomes in single studies, 14, 15, 16, 17 limited guidance exists on their application in IPD‐MA settings. In this article, we aim to provide readers with this guidance, by means of our systematic search of databases, narrative review and explanation, and an applied example. Although we focus IPD‐MA of trials, the methods we describe are also applicable to multi‐center trials.

In the next section, we provide the principles as well as several major issues of time‐to‐event analyses, that are common in not only IPD‐MA but also in single studies. In section 3 we provide details of our systematic literature search of methodology for IPD‐MA of time‐to‐event outcomes, and then a narrative review thereof follows in section 4 where we discuss the one‐ and two‐stage approaches to meta‐analysis, and in section 5 where we discuss issues in more detail. Then, in section 6 we apply several key methods of the review to a real IPD meta‐analysis of clinical trials. Finally, we give provide a discussion in section 7 and concluding remarks in section 8.

## 2 PRINCIPLES OF TIME‐TO‐EVENT ANALYSIS

The analysis of trials with a survival outcome (eg, death) typically involves statistical models that account for the time *T*_surv, *i*_ elapsed until subject *i*, *i* = 1, .., *n* developed the event of interest. We here denote the probability for subject *i* to remain event‐free for at least *t* time by the survival function *S*(*t*) = *Pr*(*T*_surv, *i*_ > *t*). A key challenge in time‐to‐event (TTE) data is that for many participants *T*_surv, *i*_ is censored to *T*_cens, *i*_, for instance due to dropout or the end of the study. This implies that for those participants *T*_surv, *i*_ > *T*_cens, *i*_. Hence, the outcome for subject *i* is typically summarized by the observed event‐free or survival time *T*_*i*_ = min(*T*_surv, *i*_, *T*_cens, *i*_) and the event status *D*_*i*_ (where *D* = 0 when censored, and *D* = 1 when the event of interest was observed to have occured). We can compare the survival times of intervention groups and control, while accounting for censoring, with a variety of regression methods.

A commonly used method for analyzing right‐censored TTE data is the Cox proportional hazards (PH) model. 18 In this semi‐parametric model the effect of the covariates is modeled parametrically, whereas the baseline is left unspecified. It is typically assumed that the ratio of the hazards for any two individuals is constant, irrespective of *t*. The hazard $h\left(t|X\right)$ for an individual with covariate vector $X^{\prime }=\left(X_{1},\ldots ,X_{k}\right)$ is given by Equation 1.1 (Table 1), where $\beta^{T}=\left(\beta_{1},\ldots ,\beta_{k}\right)$ is a vector of regression parameters. The function *h*_0_(*t*) represents the baseline hazard, which is left unspecified. 14, 15 The hazard ratio for two individuals *i* = 1, 2 is then given by $\exp\left\{\beta^{\prime }\left(X_{1}-X_{2}\right)\right\}$. For the analysis of randomized trials, X typically just contains a single covariate representing the intervention indicator (eg, *X*_*i*_ = 0 for subjects in the control arm and *X*_*i*_ = 1 for subjects in the intervention arm) such that exp(*β*) can directly be interpreted as the relative intervention effect.

**Table 1:** Models for two‐stage time‐to‐event meta‐analysis

Type	Model	Hazard function	Survival function	Ref.	No.
Proportional Hazards	General model^a^	$h_{0}\left(t\right)\exp\left(\beta^{\prime }X\right)$	$S\left(t|X\right)=S_{0}{\left(t\right)}^{\exp\left(\beta^{\prime }X\right)}$	12, 14, 162	1.1
	Exponential	$\lambda \exp\left(\beta^{\prime }X\right)$	$S\left(t|X\right)=\exp\left(-\mathrm{\lambda t}\exp\left(\beta^{\prime }X\right)\right)$	14, 16, 162	1.2
	Weibull^b^	$\lambda \nu t^{\nu -1}\exp\left(\beta^{\prime }X\right)$	$S\left(t|X\right)=\exp\left(-\lambda t^{\nu }\exp\left(\beta^{\prime }X\right)\right)$	14, 16, 162, 163	1.3
	Gompertz^c^	$\lambda \exp\left(\mathrm{\psi t}\right)\exp\left(\beta^{\prime }X\right)$	$S\left(t|X\right)=\exp\left(-\frac{\lambda }{\psi }\left(\exp\left(\mathrm{\psi t}\right)-1\right)\;\exp\left(\beta^{\prime }X\right)\right)$	14, 16, 164	1.4
Accelerated Failure Time	General model	$h_{0}\left(t\exp\left\{\beta^{\prime }X\right\}\right)\exp\left(\beta^{\prime }X\right)$	$S\left(t|X\right)=S_{0}\left(t\exp\left(\beta^{\prime }X\right)\right)$	14, 16, 89	1.5
	Weibull	$\lambda \nu t^{\nu -1}{\left(\exp\left(\beta^{\prime }X\right)\right)}^{\nu }$	$S\left(t|X\right)=\exp\left(-\lambda t^{\nu }\exp\left(\nu \beta^{\prime }X\right)\right)$	14, 16	1.6
	Log‐logistic^d^	$\frac{\varphi }{t\left\{1+t^{-\varphi }\exp\left(-\beta^{\prime }X\right)\right\}}$	$\log\frac{1-S\left(t|X\right)}{S\left(t|X\right)}=\text{φlog}\left(t\right)+\beta^{\prime }X$	89, 98, 99	1.7

a In the Cox Proportional Hazards model, the baseline hazard *h*_0_(*t*) is left unspecified.

b *ν* is a shape parameter, *λ* is a scale parameter.

c The Gompertz distribution can be generalized to the Gompertz‐Makeham distribution by adding a constant to the hazard function. 165.

d The log‐logistic model is a proportional odds model, where the *β* parameters can be interpreted as log‐odds ratios.

An important consideration is whether to include other (prognostic) covariates in the Cox PH model alongside treatment. In many time‐to‐event models, including the Cox PH model, the observed unadjusted intervention effect of a protective intervention may change over time due to covariates (ie, frailty), even if these covariates are perfectly balanced between the intervention groups. 19, 20 Frail participants will have a higher incidence rate than less frail participants. If the intervention is protective, frail participants in the intervention group will have a lower incidence rate than frail participants in a control (or an ineffective intervention) group and participants that are not frail. Over time, the proportion in the control group that is still at risk will increasingly consist of participants that are not frail, whereas this will take longer for the intervention group, thereby resulting in an imbalance in frailty. For trials with a high event rate and most frailty distributions, the unadjusted intervention effect will attenuate towards the null (hazard ratio of 1) as time progresses, which violates the proportional hazards assumption. 21 The unadjusted intervention effect is then the marginal intervention effect, 22 i.e. the average intervention effect for the population as a whole, averaged across all time‐points. Hence, it is dependent on the length of the follow‐up.

If the intention is to measure a conditional intervention effect, that is, the intervention effect for a participant with given covariate values, the observed unadjusted intervention effect is often not valid. Instead, covariates should be included in the model, to obtain a conditional intervention effect. 23, 24 Further, the adjustment for a prognostic covariate often increases the power for finding an intervention effect. 25 Alternatively, an AFT model could be used (sections 5.1 and 5.2), for which the effect of missing covariates is absorbed into the baseline parameters, leaving the unadjusted intervention effect unaffected. 21

The Cox PH model has numerous appealing properties, in particular allowing the estimation of hazard ratios for included covariates without requiring the shape of the baseline hazard to be specified. However, its implementation is not always justified. For instance, difficulties may arise when hazards are non‐proportional. Although effects to model non‐PH can be included (eg, with splines, interactions or time‐varying effects) in a Cox PH model, this usually complicates the interpretation of the estimated intervention effect. For these reasons it is often recommended to adopt a model where proportionality occurs on another scale when proportionality of hazards is violated, which is discussed in section 5.2. When absolute survival probabilities for individual participants are of primary interest, it can be useful to define a parametric function for *h*_0_(*t*), and thus to abandon Cox PH models altogether, 26, 27 which is discussed in section 5.1. Indeed, even when the focus is mainly on an intervention effect, translation of its hazard ratio to the absolute risk scale is important, which requires the baseline survival to be modelled, either parametrically or non‐parametrically. For a full overview of R packages on time‐to‐event analysis, see http://cran.r-project.org/web/views/Survival.html.

## 3 IPD META‐ANALYSIS METHODS: REVIEW

Increasingly often, IPD from multiple studies are available for analysis. This introduces new challenges and allows for different approaches for analysis, which we set out to identify. We conducted a literature review to identify scientific articles concerning statistical methods for IPD‐MA of time‐to‐event data.

### 3.1 Methods

We systematically searched through Pubmed and Web of Science using the search filters supplied in Supporting Information 1, from conception until December 31^st^, 2018. In addition, we added suggestions and performed cross‐reference checks of the obtained articles. Articles were considered eligible for inclusion if they described statistical methods for analyzing multiple or clustered individual participant data sets with a time‐to‐event outcome. Publications that met at least one of the following criteria were excluded from our review:

- Full text of the manuscript not available,
- Not published in English,
- Not a peer reviewed article,
- Application of methods without methodological focus,
- No focus on at least one of the following topics:
  - time‐to‐event outcomes,
  - IPD,
  - estimation of intervention effects,
  - meta‐analysis or analysis of clustered data.

### 3.2 Results

**Figure 1:**
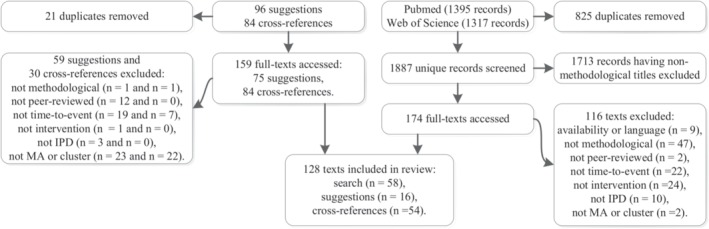
Flowchart of inclusion and exclusion of papers for review

A total of 1887 unique records were identified through our search strategy, and were deemed eligible for title and abstract screening (Figure 1). Of these, 1713 were removed during screening because the titles did not have a methodological focus. The remaining 174 records were assessed on the full‐text, of which 58 met the inclusion criteria and 116 did not. Further, a total of 159 unique records were assessed after being suggested or found through cross‐referencing. Of these, 16 suggestions and 54 cross‐references met the inclusion criteria and were included in the review. A total of 128 articles were included in the review, of which a complete list can be found in Supporting Information 3.

The core methods for analyzing TTE outcomes in IPD‐MA are described in section 4. The structure of this section was defined independent of the review, yet the description of methods therein has resulted from the review. Further, extensions to these methods, such as relaxing the proportionality of hazards assumption, modeling multiple interventions or outcomes, and methods for missing data are described in section 5.1, which was grouped according to the topics identified in the review. The review has resulted in ten key recommendations backed by references, which are summarized in Table 2.

**Table 2:** Ten Recommendations for the IPD‐MA of TTE data from Randomized Trials Examining an Intervention Effect

Recommendation	Reference
The Cox model may be the default model of choice, but proportionality of hazards	46, 166
should be tested, for example, with interaction or time‐varying effects for the intervention.	
Consider non‐PH models.	89, 92, 121, 167, 168
Account for clustering in one‐stage models, preferably by stratification of the baseline.	19, 29, 65, 67, 73, 74
Adjust for covariates measured before randomization.	25, 163, 169
Apply one‐stage models if trials are very small or the outcome very rare.	16, 28
In one‐stage models, center covariates within trials.	88
Model participant‐level interactions on the participant‐level.	45
For the intervention effect (& its interaction effects), apply random effects & investigate heterogeneity.	5, 12, 44, 78
If competing risks are present & absolute risks are of interest, apply competing risks models.	105, 108, 109, 111, 170
Multiple imputation of missing covariates must account for clustering & time‐to‐event,	136, 137, 138, 140, 143
using the event indicator and the Nelson‐Aalen cumulative hazard.	
	

## 4 DESCRIPTION OF METHODS

### 4.1 Time‐to‐event analysis in individual participant data meta‐analysis

When IPD from multiple trials are available, summary estimates for relative intervention effects can be obtained using the so‐called one‐stage or two‐stage approaches. 28 In the conceptually simpler two‐stage approach (section 4.2), the IPD from each trial is analyzed separately to produce trial‐specific estimates of relative intervention effect (eg, hazard ratios), using the same methodology in each trial (eg, Cox regression). In the second stage, estimates of intervention effect are combined into a weighted average using traditional meta‐analysis methods that ideally account for possible between‐trial heterogeneity. In the one‐stage approach (section 4.3), data from all studies are analyzed in one analysis, and a variety of methods can be used to account for clustering of participants within studies. 7, 14, 16, 29, 30 In both the one‐ and two‐stage approaches, methods to account for heterogeneity in intervention effects across studies are available (Table 3). 1, 7, 30 In the one‐stage approach, one must also decide how to model or account for heterogeneity in other parameters (such as adjustment factors or terms defining the baseline hazard). For a discussion on the choice between the one‐stage and two‐stage approaches see section 8.

**Table 3:** Methods for Modeling Heterogeneity

Baseline	Coefficients	Modeled difference between trials
Common	Common	No difference, same for every trial
Frailty	Random Effects	Proportional differences, difference between trials follows distribution
Fixed^a^	Fixed^b^	Proportional differences, estimated per trial. Same shape between trials.
Stratified		Non‐proportional differences. Estimated per trial, with different shapes.
		

a By adding trial indicators to the model.

b By adding trial indicators * variable interaction to the model.

### 4.2 Two‐stage approach

The two‐stage approach is often considered the most convenient approach for IPD meta‐analysis, as it does not necessarily require IPD to be exchanged. For instance, each trial can be analyzed separately, and only their summary statistics are combined. The approach is particularly appealing when not all trials provide IPD, as it allows reported intervention effects and their respective standard errors from non‐IPD trials to be analyzed in the second stage, together with the estimates from the IPD trials.

In the first stage, common methods for TTE analysis can be used to obtain estimates of relative intervention effect for each trial (so‐called aggregate data). For instance, when applying Cox regression (Equation 1.1), this yields the log hazard ratio estimates ${\hat{\beta }}_{j}$ and their corresponding error variance $V\left({\hat{\beta }}_{j}\right)$, for trial *j* = 1, …, *J*. Afterwards, the estimated intervention effects can be summarized by calculating a weighted average. For instance, in a so‐called common (or fixed) effect meta‐analysis it is assumed that all trials share a common intervention effect *β*_IV_, which can be derived as follows:

(1) $$\begin{array}{l} \beta_{\mathrm{IV}}=\frac{\sum_{j=1}^{J}\frac{{\hat{\beta }}_{j}}{V\left({\hat{\beta }}_{j}\right)}}{\sum_{j=1}^{J}\frac{1}{V{\hat{\beta }}_{j})}}\\ V\left(\beta_{\mathrm{IV}}\right)=\frac{1}{\sum_{j=1}^{J}\frac{1}{V\left({\hat{\beta }}_{j}\right)}} \end{array}$$

where *V* is the variance. Hereby, it is assumed that the within‐trial variances $V\left({\hat{\beta }}_{j}\right)$ are known (ie, estimated without uncertainty). The common effect meta‐analysis model can also be formulated as follows:

(2) $${\hat{\beta }}_{j}\sim N\left(\beta_{\mathrm{IV}},V\left({\hat{\beta }}_{j}\right)\right)$$

If certain trials provide no IPD, but the intervention effect and its variance are available in the literature, these can be included in the second stage of the two‐stage framework, 31 provided that the models in the first stage are specified the same. If a trial has a small sample size, the Maximum Likelihood estimator of the intervention effect can be affected by small sample bias. 32 Worse still, if considerable censoring is present, the likelihood may be monotone and the Maximum Likelihood may be inestimable, depending on the intervention and covariate distributions. 33 This can be resolved by applying Firth's correction to the likelihood in the first stage, 32, 33 or by opting for a one‐stage model instead.

The assumption that an intervention effect is common across trials is often unrealistic, as trials are often affected by between‐trial heterogeneity. 34, 35 This heterogeneity may, for instance, appear when participant‐level covariates interact with the intervention effect (ie, effect modification), when small sample bias is present in some estimates of the intervention effect, or when aggregate data are based on invalid modeling assumptions (eg, in the presence of non‐proportional hazards, non‐PH). For time‐to‐event analysis, between‐trial heterogeneity may also arise due to selection effects. In particular, participants who are more frail and therefore more susceptible to the outcome, are no longer at risk after having an event. Therefore, over time, the most frail participants are removed from the risk set, whereas the less frail participants remain at risk (see section 2). 14, 19, 23, 36 This, in turn, may lead to different intervention effects across trials if the follow‐up length differs across trials. For these reasons, in the two‐stage approach it is generally recommended to adopt a random effects meta‐analysis model, which is typically specified as:

(3) $$\begin{array}{ll} {\hat{\beta }}_{j} & \sim N\left(\beta_{j},V\left({\hat{\beta }}_{j}\right)\right)\\ \beta_{j} & \sim N\left(\beta_{\mathrm{RE}},\tau^{2}\right) \end{array}$$

In contrast to common effect models, random effects models allow for differences in ${\hat{\beta }}_{j}$ due to sampling error *within* studies (reflected by $V\left({\hat{\beta }}_{j}\right)$) and due to heterogeneity in the true intervention effects *β*_*j*_ *across* studies (reflected by *τ*^2^). Estimates for *β*_RE_ can thus be interpreted as the average intervention effect across studies. A confidence interval for ${\hat{\beta }}_{\mathrm{RE}}$ is traditionally constructed as ${\hat{\beta }}_{\mathrm{RE}}\pm z_{1-\alpha /2}\sqrt[{}]{V\left({\hat{\beta }}_{\mathrm{RE}}\right)},$ where *z*_1 − *α*/2_ is the upper *α*/2 quantile of the standard normal distribution. 37 To account for the uncertainty in *τ*^2^ and thereby improve the coverage of the interval, the Hartung‐Knapp approach to confidence intervals is given by ${\hat{\beta }}_{\mathrm{RE}}\pm t_{J-1,1-\alpha /2}\sqrt[{}]{V_{\mathrm{HK}}\left({\hat{\beta }}_{\mathrm{RE}}\right)}$, where *t*_*J* − 1, 1 − *α*/2_ is the upper *α*/2 quantile of a *t*‐distribution with *J* ‐ 1° of freedom, and $V_{\mathrm{HK}}\left({\hat{\beta }}_{\mathrm{RE}}\right)$ is a modified variance estimate. 38, 39, 40, 41, 42

#### 4.2.1 Heterogeneity of the intervention effect in the two‐stage approach

Statistical heterogeneity in the intervention effect can be recognized by $\hat{\tau }>0$. The influence of heterogeneity on intervention effects may be explored by constructing a prediction interval, which estimates the interval of the likely intervention effect in a (new) individual trial and can be calculated approximately as follows 43, 44:

(4) $${\hat{\beta }}_{\mathrm{RE}}\pm t_{J-k,1-\alpha /2}\sqrt[{}]{{\hat{\tau }}^{2}+V\left({\hat{\beta }}_{\mathrm{RE}}\right)},$$

where ${\hat{\beta }}_{\mathrm{RE}}$ is an estimate of *β*_RE_ and $V\left({\hat{\beta }}_{\mathrm{RE}}\right)$ its variance. Typically the *t*_*J* − 2, 1 − *α*/2_ quantile is used here, although similar to the confidence interval there is no consensus on the distribution and its degrees of freedom. 43, 44 When random effects models indicate the presence of important statistical heterogeneity (ie, $\hat{\tau }>0$, or a wide prediction interval) of the intervention effect, the interpretation of the overall summary estimate, ${\hat{\beta }}_{\mathrm{RE}}$, may become difficult or meaningless. Therefore, it is often helpful to identify sources of heterogeneity in intervention effect (see Table 4). 12 This can, for instance, be achieved by assessing the relation between relevant trial‐level covariates (eg, level of blinding, or dosage) and the trial effect estimates, also known as meta‐regression. 45

**Table 4:** Potential sources of Heterogeneity in Time‐to‐event Meta‐Analysis

Source	Solutions	Reference
Non PH + Differences in follow‐up time	Interaction terms Model effect(s) as time‐varying, use splines Use a different model (eg, AFT)	45, 88, 171 83, 91 27, 83, 92, 103, 167, 168
Difference in case‐mix	Include covariates/prognostic factors AFT model	36, 163 92, 163, 168
Selective dropout or competing risk	Model dropout or competing risk	108, 111, 170, 172
Small sample bias in some studies	Bias correction One‐stage MA Arcsine transform (for two‐stage MA)	32 7, 28, 172, 173 172

PH: Proportional Hazards; AFT: Accelerated Failure Time; MA: Meta‐Analysis. Heterogeneity can be diagnosed by applying frailty and/or random effects terms. 13, 29, 78 If heterogeneity remains, for example, due to differences in study protocols, stratification of baseline hazard/frailty and/or random effects terms must be applied. 24, 36.

When patient‐level associations with treatment effect are of interest, it is better to model interactions between participant‐level characteristics (eg, participant age) on the participant level. In the two‐stage approach, the statistical interaction between the relevant covariate and intervention are first estimated separately in each trial, and then the resulting coefficients are meta‐analyzed using traditional meta‐analysis models. 34, 46 When the intervention effect changes over time, differences in follow‐up time between trials will lead to heterogeneous estimates of intervention effect across trials, if unaccounted for. This heterogeneity of intervention effects can be quantified with random‐effects meta‐analysis, but would preferably be modeled directly (section 5.2).

#### 4.2.2 Estimation

A commonly used approach to estimate the heterogeneity from the random effects model (Equation 3), is to use the method of moments by DerSimonian and Laird (DL). 47 This estimator is biased downwards when the true heterogeneity is moderate or high and sample sizes are low, as the variance estimates are assumed to be known and fixed, 48 leading many researchers to suggest alternatives, the most important of which are mentioned here. The two‐step Paule‐Mandel method is similar to DL, but iteratively estimates the study weights, and has reduced bias for high values of *τ*. Another alternative is the Maximum Likelihood (ML) estimator. Although the MSE of the ML estimator for *τ* is small, it is very biased when *τ* is large and the included studies are small. 49 The Restricted Maximum Likelihood (REML) estimator yields less biased estimates of *τ* and has relatively low MSE. 50, 51, 52 Therefore, REML and the two‐step Paule‐Mandel method are the recommended estimators for *τ*. 48, 52

As there may be considerable uncertainty in the heterogeneity estimate regardless of which estimator is used, 52 it is recommended to report a confidence interval for the heterogeneity as well. 53 This may be estimated with the Q‐profile method 41, 54 or the generalised Cochran between‐study variance method. 49 Further, it should be noted that when fewer than 10 trials are included in the meta‐analysis, or when trials are small or the outcome rare, no currently available method can reliably estimate the heterogeneity. 52

Even though estimates for heterogeneity in meta‐analysis tend to be biased in many situations, this barely biases the summary effect estimate, unless there are very few events. 52 The confidence intervals of the summary effect can be constructed by applying the Hartung‐Knapp‐Sidik‐Jonkman HKSJ method for confidence intervals, 55, 56 which had good coverage in simulations for a minimum of two studies, unless the number of events was very low. 52, 57 This may be corrected by applying a modification that ensures that the confidence intervals are at least as wide as a fixed‐effects meta‐analysis confidence interval. 52 Hence, it is currently recommended to apply a random effects model estimated with REML or two‐step Paule‐Mandel, and to use the HKSJ method for confidence intervals. 52 Alternatively, Bayesian random‐effects models may be used. However, in the simulation studies discussed here either aggregate data or non time‐to‐event IPD were generated, which is a concern considering that it has been suggested that the performance of the estimators may be related to the type of outcome. 49 For a comprehensive overview of meta‐analysis estimators see 49, 58, 59, for a comparison of their performance see 48, 52, for an overview of software see 49 as well as the two recent packages admetan and ipdmetan, 60 and for an up‐to‐date overview of R packages see http://cran.r-project.org/web/views/MetaAnalysis.html.

### 4.3 One‐stage approach

#### 4.3.1 Accounting for clustering

When applying the one‐stage approach, within‐trial and between‐trial relationships are estimated simultaneously, which can give a more complete understanding of the data. 13 As is the case for two‐stage meta‐analysis, a one‐stage meta‐analysis must account for clustering (Table 3). 29, 30 Participants in different studies may differ on unmeasured covariates, which will lead to a biased estimate of the conditional (ie, for a participant with given covariate values) intervention effect regardless of balance of these covariates between intervention groups, if not adjusted for (section 2). 20 Whereas the two‐stage approach naturally deals with this by estimating separate baseline hazards for the different studies, in the one‐stage approach we can use stratification (section 4.3.2), frailty models (section 4.3.3) or marginal models (section 4.3.4).

#### 4.3.2 Stratified models

A commonly used approach is to apply a Cox model with stratified baseline hazards but a common intervention effect (Equation 5.1, Table 5). 12, 16, 61, 62 This allows the shapes of the baseline hazards to vary between trials, whereas the hazards of the different intervention groups are assumed to be proportional within trials, and gives a single estimate of overall intervention effect. When the sample sizes per trial are very small and many trials are included, the stratification of baselines is less efficient than the use of frailty terms, 16 though it also requires fewer assumptions as it fully accounts for any differences in baselines between trials. For the meta‐analysis of trials that are each powered to detect a clinically significant intervention effect this should not be an issue, thereby making the stratification of the baseline the preferred model specification.

**Table 5:** Models for one‐stage time‐to‐event meta‐analysis

Type	Model	Hazard function	Survival function	Ref.	No.
Proportional Hazards	Stratified baseline	$h_{0j}\left(t\right)\exp\left(\beta^{\prime }X_{j}\right)$	$S_{j}\left(t|X_{j}\right)=S_{0j}{\left(t\right)}^{\exp\left(\beta^{\prime }X_{j}\right)}$	12, 14, 16, 18, 174	5.1
	Shared frailty	$h_{0}\left(t\right)\eta_{j}\exp\left(\beta^{\prime }X_{j}\right)\;\text{where}\;\eta_{j}\sim \text{Gamma}\left(\theta \right)\;\text{or}\;\log\left(\eta_{j}\right)\sim \text{Normal}\left(0,\tau^{2}\right)$	$S_{j}\left(t|X_{j}\right)=S_{0}{\left(t\right)}^{\eta_{j}\exp\left(\beta^{\prime }X_{j}\right)}$	12, 14, 16, 19, 174	5.2
	Random effects	$h_{0}\left(t\right)\exp\left(\beta^{\prime }X_{j}+b_{j}^{\prime }Z_{j}\right)\;\text{where}\;b_{j}\sim \mathrm{MVN}\left(0,\sum \right)$	$S_{j}\left(t|X_{j}\right)=S_{0}{\left(t\right)}^{\exp\left(\beta^{\prime }X_{j}+b_{j}^{\prime }Z_{j}\right)}$	13, 29, 79, 80, 83, 85, 174	5.3
					
Accelerated Failure Time	Stratified baseline	$h_{0j}\left(t\exp\left\{\beta^{\prime }X\right\}\right)\exp\left(\beta^{\prime }X\right)$	$S_{j}\left(t|X_{j}\right)=S_{0j}\left(t\exp\left(\beta^{\prime }X\right)\right)$	16	5.4
	Shared frailty	$h_{0}\left(t\eta_{j}\exp\left\{\beta^{\prime }X\right\}\right)\eta_{j}\exp\left(\beta^{\prime }X\right)$ where *η*_*j*_~Gamma(*θ*) or *log*(*η*_*j*_)~Normal(0, *τ*^2^)	$S_{j}\left(t|X_{j}\right)=S_{0}\left(t\eta_{j}\exp\left(\beta^{\prime }X\right)\right)$	16, 81, 83, 168	5.5
	Random effects	$h_{0}\left(t\exp\left\{\beta^{\prime }X_{j}+b_{j}^{\prime }Z_{j}\right\}\right)\exp\left(\beta^{\prime }X_{j}+b_{j}^{\prime }Z_{j}\right)\;\text{where}\;b_{j}\sim \mathrm{MVN}\left(0,\sum \right)$	$S_{j}\left(t|X_{j},Z_{j}\right)=S_{0}\left(t\exp\left(\beta^{\prime }X_{j}+b_{j}^{\prime }Z_{j}\right)\right)$	16, 81, 83, 168	5.6
					

In the Cox Proportional Hazards model, the baseline hazard *h*_0_(*t*) is left unspecified. For the baseline hazard of the parametric models, see Table 1.

#### 4.3.3 Frailty models

Rather than stratifying the baseline hazard across the trials, it is possible to model their distribution through frailty terms. A frailty term is a random parameter (ie, random intercept) within the baseline hazard function that is assumed to follow a specified distribution and thereby allows for differences in baseline rate between (groups of) participants that are a result of unmeasured covariates. Shared frailty models (Equation 5.2, Table 5) are designed to account for these differences in unmeasured covariates between trials. Therefore, the assumption in a frailty model is that the baseline hazards in each study have the same shape but a different magnitude. The estimated intervention effect is then to be interpreted relative to other participants in the same trial with the same frailty and covariates. If the baseline hazard of this model is left unspecified, this leads to the Cox PH model with random trial intercept. 12, 14 When data from multiple multi‐center studies are combined, nested frailty models can be applied. 63

It is common to assume a gamma distribution for the frailty, for mathematical or computational reasons, 14, 24 or a normal distribution for the log‐frailty, as this bears similarity to the generalized linear mixed effects model, 14, 64 though many other distributions including the inverse Gaussian, positive stable, and compound Poisson are possible. 14, 16, 17 Previous studies have demonstrated that the gamma frailty model appears to be fairly robust against misspecification of the frailty distribution, 65, 66 that it describes the frailty of survivors for a large class of hazard models, 24 and that it can have more power than a stratified model. 16, 66, 67 Therefore, frailty models are generally recommended when the number of participants per trial is very low. Yet, when the number of participants per trial is large, as is often the case in meta‐analysis when individual trials are designed to have sufficient power to test for an intervention effect, the frailty and stratification approaches will usually yield similar results, given that the assumptions are met.

When a frailty is applied to the baseline hazard, the median hazard ratio (MHR) can be used to evaluate the meaning of this frailty in the context of the different studies. 68, 69, 70 The MHR is the median relative difference in the hazard of the occurrence of the outcome when comparing identical participants from two randomly selected studies ordered by hazard. When a log‐normal distribution is assumed for the frailty, the Median Hazard Ratio (MHR) can be computed as $\exp\left\{\sqrt[{}]{2\sigma^{2}}\Phi^{-1}\left(0.75\right)\right\}$, where Φ^−1^ is the inverse of the standard normal distribution. 69, 70

#### 4.3.4 Marginal models

In the analysis of clustered data, such as IPD from different studies, where the interest lies in the average intervention effect for the target population as a whole, we may use marginal models. In such models the dependence between participants from the same trial is not modeled explicitly but standard errors are adjusted for it. 71, 72 Intervention effects are interpreted as relative to participants drawn randomly from the entire target population from which the participants are considered to be sampled. 73 When the interest lies in the intervention effect of participants in the individual studies or in the causes of heterogeneity of intervention effects across studies or subgroups, as in an IPD‐MA often is the case, conditional models are needed. 74

#### 4.3.5 Estimation

Maximum Likelihood (ML) estimates of the mixed effects Cox model may be obtained with a Newton–Raphson procedure, 75 with penalization methods by constraining the frailty terms with a penalty, 76, 77, 78 by expectation‐maximisation, 79 or by expectation‐maximisation and penalization. 80

Further, residual maximum likelihood (REML) estimates of the mixed effects Cox model can be obtained with a Newton–Raphson procedure, 12, 13, 75 or with penalization methods by constraining the frailty terms with a penalty. 76, 77 As the penalized method does not take uncertainty of *τ*^2^ into account, it has been suggested that it produces less precise estimates of the intervention effect. 62 However, comparative evidence is currently lacking.

Alternatively, the mixed effects Cox model can be estimated with a poisson model, 81 where the time‐scale is split into intervals defined by event times. 82 Mixed effects parametric models can be estimated with Maximum Likelihood by adaptive Gauss‐Hermite quadrature. 83 Mixed effects Weibull models can also be estimated with REML. 81

The Bayesian framework allows for the estimation of a wide range of time‐to‐event models. For instance, the Cox random effects model can be estimated using Bayesian methods. 16, 84, 85 A random trial effect and an intervention by trial interaction may be evaluated simultaneously in a Bayesian Cox PH model. 86 For a discussion of commensurate priors for incorporating between‐trial variability in a Bayesian meta‐analysis, see 87. Finally, an overview of software for the estimation of one‐stage time‐to‐event models is given in Table 6.

**Table 6:** Software for One‐stage Time‐to‐event Models

Program	Package/method	Description	Code in	Mentioned in
R, S‐Plus	‐	Random effects Cox model	80	
	survival	Cox and parametric time‐to‐event models.	66, 77, 175, 176	
		Stratified, frailty and marginal specifications		
	coxme	Mixed effects Cox models		
	frailtypack	Cox and parametric random effects and stratified models.		63, 78, 111
		Correlated random effects. Competing events. Joint nested frailty models.		
	SemiCompRisks	Bayesian and frequentist random effects parametric and		111
		semi‐parametric models for competing events.		
	parfm	Parametric frailty models		
	PenCoxFrail	Regularized Cox frailty models		
	mexhaz	Flexible (excess) hazard regression models,		
		non‐proportional effects, and random effects		
	dynfrail	Semiparametric dynamic frailty models		
	frailtyEM	Frailty models with semi‐parametric baseline hazard, recurrent events		
	joineR	Joint random effects models of repeated measurements & time‐to‐event		
	joint.Cox	Joint frailty‐copula models with smoothing splines		
	JointModel	Joint model for longitudinal and time‐to‐event outcomes		
	joineRML	Joint time‐to‐event and multiple continuous longitudinal outcomes		
	rstanarm	Joint model for hierarchical longitudinal and time‐to‐event data	131	
	surrosurv	Time‐to‐event surrogate endpoints models	177	
				
SAS	PHREG	Cox models, including stratification or frailty	66, 175	178
	NLMIXED	Mixed effects parametric survival models		179
		Joint model for recurrent events and semi‐competing risk	112	
	GENMOD	Poisson regression, marginal models		178
				
Stata	stcox	Cox model, stratified and frailty specifications.		
	stmixed	Flexible parametric time‐to‐event models with mixed effects		7, 83
	xtmepoisson	Mixed effects Poisson regression	82	
				
WinBUGS, OpenBUGS, JAGS	‐	Bayesian mixed effects models,	67, 82, 175	
	‐	IPD network meta‐analysis	118, 121	
				
MLwiN	‐	Mixed effects time‐to‐event models		7, 180
				
The Survival Kit	‐	Bayesian mixed effect time‐to‐event models		86
				

#### 4.3.6 Heterogeneity of the intervention effect in the one‐stage approach

Similar to the two‐stage approach, we may expect heterogeneity of the intervention effect in the one‐stage approach, which makes the common effects assumption untenable. As such, it is also recommended for one‐stage models to assume random effects (Equation 5.3, Table 5), 79 and to investigate the causes of this heterogeneity, if present. 12 One possible cause of heterogeneity of the intervention effect is effect modification (ie, interaction) at the individual level, which can be investigated by adding an interaction term in the one‐stage model. 29 Crucially, when including such an interaction term (eg, an intervention‐covariate interaction) in the one‐stage approach, special care must be taken to avoid the amalgamation of within‐ and across‐trial information, as this may lead to ecological bias. This can be achieved by centering the covariates by their mean values within trials, such that the interaction estimate is then only based on within‐trial information. 88 To improve the estimation of between‐study variance and the coverage of confidence intervals, the intervention variable can be centered within studies as well. To further prevent the borrowing of information across studies that may affect the estimate of the intervention effect in the one‐stage approach, a covariate by trial indicator interaction can be included. This stratifies the covariates effects as it allows covariate effects to be estimated separately for each study (see Table 3).

When there are differences in follow‐up time between trials and the intervention effect changes over time, the estimated intervention effects (as quantified by random effects) will be different per trial. If this is unaccounted for, this will lead to heterogeneity of the intervention effect. This can then be investigated by modeling the effect as time‐dependent (section 5.2).

In the two‐stage approach the influence of trial‐level characteristics on the intervention effect can be estimated with meta‐regression in the second stage. In the one‐stage approach it is possible to simultaneously estimate the heterogeneity of baseline rate of the participants within different studies, the heterogeneity of intervention effects and their correlation. 78

## 5 EXTENSIONS

### 5.1 Modeling the baseline hazard function

Whereas the Cox PH model leaves the baseline hazard unspecified, we may apply a parametric model by specifying a baseline hazard (Table 1), either in the first stage of the two‐stage approach, or within the one‐stage approach. To allow for flexible shapes of the baseline hazard, we can apply spline functions. Particularly the approach of Royston and Parmar is useful, where the baseline cumulative hazard is modelled using restricted cubic splines, 89 and which has been extended to allow for random effects. 83

Parametric models are especially suitable when absolute (rather than relative) risks for individual subjects (rather than for subpopulations) are of primary interest. It leads to smooth predicted survival curves and is well suited to deal with non‐proportionality of hazards. For instance, researchers increasingly often aim to develop prediction models that can assess individual intervention benefits (or harms). 90 Most simply, one can specify an exponential (Equation 1.2) or a Weibull (Equation 1.3) distribution within the proportional hazards framework. The exponential distribution assumes a constant rate over time, whereas the Weibull distribution (a generalization of the exponential distribution) allows for accelerated failure times (AFT). 14 Other (but less common) generalizations of the exponential distribution that can be used for modeling the baseline hazard are the Gompertz, gamma, and piecewise constant distributions. 14, 83 Further, the log‐logistic, log‐normal and generalized gamma distributions may be used. 83, 89 Unlike PH models, the estimate of an intervention effect in AFT models is unaffected by unmeasured prognostic covariates. 21 Also in one‐stage models a wide range of distributions for parametric PH and AFT models is available. 83

### 5.2 Modeling non‐proportional hazards

For short trials with a low event rate the proportionality of hazards across time may be reasonable (ie, the hazard ratio for the intervention effect may be assumed constant over time), but as the number of events in different intervention groups diverges a selection of participants remains in the trial for whom proportionality in the unadjusted intervention effect is not realistic. 91, 92 If an intervention is protective, frail participants in the intervention group will be better protected against the outcome than frail participants in the control group. Hence, the proportion of frail participants at risk will decrease more quickly in the control group than in the intervention group. To account for this issue within studies we can include covariates in the model, whereas we can use a frailty model to account for this issue between studies.

Non‐proportionality of hazards may also be present due to the intervention effect truly being dependent on time. For instance, an intervention (such as surgery or chemo‐therapy) may cause an increased risk of a negative outcome at first, but have a protective effect in the long run. This can be modeled by an interaction effect between the intervention (or a covariate) and time 18 in the one‐stage approach or in the first stage of the two‐stage approach. To allow for flexible shapes of this time‐dependent effect, fractional polynomials or splines can be applied. 93, 94, 95

Two methods have been developed for combining fractional polynomials or splines in the two‐stage approach. The meta curve method directly meta‐analyzes the curves estimated in the first stage. Though, this requires setting a reference level which may have an impact on the results. Alternatively, by using multivariate meta‐analysis (section 5.3) the coefficients can be combined. This method only works when the same polynomials or splines have been fitted in each study, but that is not an issue when IPD are available. 96

Alternatively, non‐PH can sometimes be handled more naturally with models that assume proportionality on another scale. 89, 92 For instance, an intervention might temporarily reduce the hazards, but as time progresses and the effect wears off, hazards converge and thereby violate the proportional hazards assumption. This can be modeled with a proportional odds regression model such as the log‐logistic (Equation 1.7, Table 1), which assumes that covariates have a constant additive effect on the log odds of survival. 27, 97, 98, 99 In this model, the modeled hazard ratio naturally approaches 1 over time, whereas the odds remain proportional. 98

As the implementation of TTE models with non‐proportional hazards (eg, with splines) may complicate the interpretation of regression parameters, alternate effect measures have been proposed to summarize intervention effects (Table 7). For instance, the restricted mean survival time (RMST, Equation 7.3) until time t^*^ represents the area under the survival curve until time t^*^. 100, 101, 102 The RMST can thus be calculated for different intervention groups, and subsequently be subtracted to assess the intervention effect. This difference represents the expected gain (or loss) in survival until time t^*^ for the intervention group, as compared to the control group. An advantage is that it provides a clinically meaningful summary of the survival differences between intervention groups.

**Table 7:** Effect Measures for Time‐to‐Event Analysis

Measure	Definition		Ref.	No.
Hazard ratio	$\frac{\lambda \left(t|X_{1}\right)}{\lambda \left(t|X_{0}\right)},$	$\lambda \left(t|X_{k}\right)=-\frac{d\ln\left(S\left(t|X_{k}\right)\right)}{\mathrm{dt}}=\frac{f\left(t|X_{k}\right)}{S\left(t|X_{k}\right)}$	14, 15	7.1
Odds ratio	$\frac{O\left(t|X_{1}\right)}{O\left(t|X_{0}\right)},$	$O\left(t|X_{k}\right)=\frac{1-S\left(t|X_{k}\right)}{S\left(t|X_{k}\right)}$	27	7.2
RMSTD(t^*^)	RMST_1_(t^*^) − RMST_0_(t^*^),	$\text{RMST}\left(t^{*}\right)=\int_{0}^{t^{*}}S_{k}\left(t\right)\mathrm{dt}$	100, 102	7.3
Percentile Ratio	$q_{k}=\frac{k^{\mathrm{th}}\text{percentileofdistforgroupA}}{k^{\mathrm{th}}\text{percentileofdistforgroupB}}$		103	7.4
				

RMST = Restricted Mean Survival Time, D = Difference.

The percentile ratio, an effect measure alternative to the more common hazard ratio, was suggested by to make the interpretation of survival models more straightforward. 103 Briefly, the percentile ratio for an intervention is defined as the expected ratio for the time at which a certain fraction (given as ‘k') of the participants will have an event in the intervention group as compared to the control group (Equation 7.4). The percentile ratio is easiest to interpret for AFT models, as the percentile ratio does not depend on the percentile chosen in such models and always equals the acceleration factor. Two‐stage MA methods for the percentile‐ratio have also been developed. 104

### 5.3 Modeling multiple outcomes

Throughout this manuscript, we have assumed that each patient in each trail is at risk of having a single type of event (ie, the outcome of interest, for example, all‐cause mortality), until censoring takes place. Alternatively, patients may be at risk for different events, where one event (eg, death) prevents the patient from having another event (eg, liver failure or stroke). Unlike the survival function, relative intervention effects can then still be assessed by modeling cause‐specific hazards, which involves the modeling of the time to each type of event in a separate model, where all alternative types of event are coded as censoring. 105, 106 It is vital to do this for every type of event, to gain a full understanding of the relative intervention effect with respect to competing events. 105 Whereas for all‐cause‐mortality there is a direct relation between the hazard and the survival curve, when modeling cause‐specific hazards this is not the case, 107 meaning that this approach does not have a direct interpretation in terms of absolute survival probabilities for the outcome of interest. 108 Only when independence of the event of interest and the competing event can be assumed, the survival function can be estimated by recoding the competing outcome as censoring, though this assumption is often not realistic. 109

Therefore, when prediction of the average time‐to‐event per intervention group is wanted, competing events must be modeled using more complex survival models (for an introduction see 105, 110). In the two‐stage approach, this can be analyzed with competing risk models in the first stage, whereas Bayesian hierarchical competing risk models have been developed for the one‐stage approach, 111 which may also model recurrent events jointly with the competing risk. 112 Further, multi‐state models can be used to model transitions to intermediate events. 113

When multiple outcomes that do not compete are available across trials, these can be assessed jointly in the two‐stage framework to improve the efficiency of the analyses. 114, 115 For instance, outcomes may have been assessed at multiple follow‐up times, or be defined for multiple endpoints. In the first stage, estimates of the intervention effects and variances are obtained for each outcome in each trial. Bootstrapping is used to obtain the covariance between intervention effects for each pair of outcomes in the same trial. 115 In the second stage, the vectors of estimates (and matrices of variances and covariances) are synthesised using a multivariate meta‐analysis model in the second stage. Hence, multivariate meta‐analysis is particularly relevant to address outcomes or time‐points in the IPD from some trials.

### 5.4 Modeling multiple interventions

The concepts of multivariate meta‐analysis can also be used to compare more than two interventions. In a so‐called network meta‐analysis (NMA), direct and indirect evidence about the difference in effect of two or more treatments is combined across trials, to summarize the relative effects of all available interventions. This may improve precision of the intervention estimates and allows for comparison of interventions that have not been compared head‐to‐head. This method uses direct evidence (intervention effects estimated within trials) and indirect evidence (intervention effects estimated across trials), by assuming that both sources of evidence are exchangeable. 116, 117. When direct and indirect evidence disagree, the network is said to be inconsistent and may be prone to bias or may cause heterogeneity of the estimated intervention effects. Such inconsistency can be caused by effect modification, which can be addressed by modelling interactions between the intervention and patient‐level covariates. 118

In the two‐stage approach, an appropriate (eg, Cox) survival model is first estimated in each trial, possibly adjusting for relevant prognostic factors and effect modifiers. Corresponding effect estimates (eg, log hazard ratios) can then be pooled using traditional NMA methods. 116 In the one‐stage approach, time‐to‐event NMA models can be estimated using Bayesian hierarchical models. 119, 120 Also, Bayesian one‐stage IPD‐NMA Royston‐Parmar models have been implemented. 121

### 5.5 Surrogate endpoints

Trials for measuring intervention efficacy tend to be expensive and require a lengthy follow‐up to observe the clinical outcome. The cost and duration of a trial may be reduced if a more readily available outcome can be used. Validated surrogate endpoints can be used instead when the surrogate is well known or likely to predict clinical outcome. 122 These surrogate endpoints are to be validated on the trial and the participant level, where IPD form multiple trials are preferred. 123, 124 When response to intervention is used to predict survival, response must be modeled as a time‐dependent covariate or a landmarking method must be used. 125 Alternatively, a joint model with the survival outcome and a continuous surrogate or a dichotomous surrogate can be used. 126, 127 For an overview and comparison of the performance of measures of surrogacy, see 128, 129. When few trials are available, the trial level surrogacy cannot reliably be estimated using AD alone. However, surrogacy can sometimes be estimated on the center level by splitting multi‐center data by center. 124, 130 This requires IPD when center specific parameter estimates are not available. For a recent overview of methods for estimating surrogacy, see 130. To include a surrogate directly in the modeling of the outcome, a joint model can be used. 126, 127 For the one‐stage approach, joint models with up to three levels have also been developed. 131

### 5.6 Missing data

In a meta‐analysis of survival data, several types of missing data may occur. It is possible, for instance, that not all studies provide IPD and thus that only AD are available for some of the studies. In such cases, it is recommended to combine the available IPD and AD, as otherwise estimated intervention effects may be prone to (data availability) bias and overly large standard errors. 132 Including AD in a two‐stage meta‐analysis approach is fairly straightforward, provided that the model used for generating the AD is compatible with the models for analyzing the available IPD. It is also possible to directly combine IPD and AD using a one‐stage meta‐analysis, although this requires more advanced models, such as Bayesian hierarchical regression. 133

Another common type of missing data occurs when events of individual subjects are censored, for example, due to loss of follow‐up. Survival models such as the Cox PH model and the AFT model readily account for this censoring, provided that it is not related to the outcome, conditional on any participant‐level characteristics in the model (ie, non‐informative). When the assumption of independent censoring is challenged, its implications can be evaluated by adopting multiple imputation methods. 134

Finally, it is possible that subject‐level covariates are missing for one or more studies. Although participant covariates are not commonly used when estimating relative intervention effects from RCTs, they are crucial in IPD‐MA of time‐to‐event data because of selection differences across trials (see section 2). When relevant participant‐level covariates are missing for some trial participants, it is generally recommended to apply multiple imputation. 135 Hereby, researchers should adjust for the event indicator and the Nelson‐Aalen estimator of the cumulative hazard, 136, 137 and also account for the presence of clustering. The latter can be achieved by adopting imputation models with mixed effects, which also facilitates imputation of covariates that have not been measured in one or more studies. 138, 139, 140, 141, 142

Although the assumptions needed for multiple imputation cannot always be tested or may not always be met, several simulation studies have shown that its use is usually superior to complete‐case analysis or the use of missing data indicators. 143 However, caution is still warranted when analyzing imputed data sets from IPD‐MA, as in the presence of between‐trial heterogeneity these are inherently prone to some degree of incompatibility with the data generation mechanism. 141, 144 Further, because IPD‐MA can only adjust for measured covariates and may therefore still be affected by unmeasured covariates, clustering of participants within trials should still be accounted for (section 4.3.1). 14

## 6 APPLIED EXAMPLE

The efficacy of carbamazepine (CBZ) and valproate (VP) as interventions for epileptic seizures was compared in a systematic review and IPD‐MA of RCTs. 145 IPD were obtained for a total of 1225 participants from five trials. In all these trials, one of the outcomes of interest was time to first epileptic seizure since randomization. Also, measured covariates were age at randomization, sex, type of epilepsy (partial‐onset or generalized‐onset), and the number of epileptic seizures before randomization. For illustrative purposes, we only consider the type of epilepsy. We use the coxme package of the R software, 146, 147 to fit the mixed effects Cox PH model. Our code is given in Supporting Information 2.

As the two‐stage method has been described extensively (see 104, 148) we shall restrict our analyses to illustrate some key one‐stage methods. First, to evaluate the relative effects of CBZ and VP, we adopted a Cox model, as this leaves the baseline hazard unspecified. We apply a one‐stage model (Equation 5.2) with a log‐normal frailty and random effects for the intervention estimated with penalized partial likelihood to account for the clustering of participants within trials and to allow for heterogeneous intervention effects across trials, respectively. We find no evidence against the hypothesis that the interventions are equally effective, with a summary hazard ratio of 1.08 for valproate (95 % Confidence Interval (CI) : 0.92 to 1.27, *p* = .37), vs the referent, carbamazepine.

**Figure 2:**
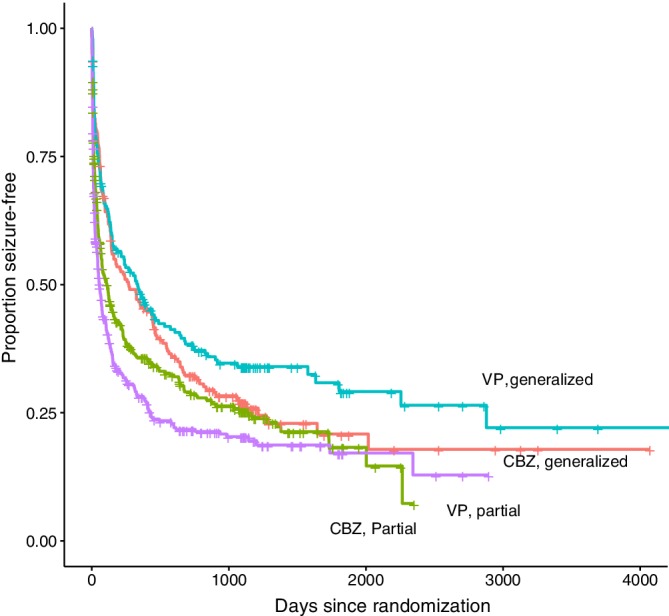
Kaplan–Meier plot of Generalized and Partial Epileptic Seizure Patients Treated with Carbamazapine (CBZ) or Valproate (VP) [Colour figure can be viewed at http://wileyonlinelibrary.com]

In the analysis of the effect of the intervention on the time to first epileptic seizure, we observed some statistical heterogeneity of the intervention effect. The SDs of the random intercept (ie, frailty) and drug effect (ie, random effect) equaled 0.139 and 0.099, respectively. In other words, the log hazard ratio of valproate vs carbamazapine varied with a SD of.099 between trials. This random effect of the interventions translated to a Median Hazard Ratio (MHR) of 1.10, meaning that the median relative change in the effect on time‐to first epileptic seizure when comparing two identical participants from two randomly selected different trials that were ordered by intervention effect was 1.10, calculated as $\exp\left\{\sqrt[{}]{2}0.099\Phi^{-1}\left(0.75\right)\right\}$ (see section 4.3.6). In order to explain this heterogeneity in intervention effect, we added covariates and intervention‐covariate interactions to the model (Figure 2). Partial epilepsy (vs generalized) was associated with a higher hazard rate (*β* = 1.63, 95% CI: 1.38 to 1.92, Table 8), meaning that we have found evidence that epilepsy type is a prognostic factor of time to first epileptic seizure. However, we were unable to find evidence that epilepsy type interacted with the intervention (*β* = 1.36, 95% CI: 0.97 to 1.89), though it should be noted that the upper bound of the CI did not exclude clinically significant effects. We note that we obtained somewhat different results than the Cochrane review, 149 as we have used a different method for analysis. Further, the low power for tests for interaction effects is a notorious issue.

**Table 8:** Intervention, Covariates and Intervention‐Covariate Interactions in a Multivariable Mixed Effects Cox Model

Variable	Variable Type	HR	95% CI	*p*
VP (vs CBZ)	Intervention	1.05	0.86 to 1.28	0.65
Partial epilepsy (vs generalized), centered^a^	Individual‐level covariate	1.63	1.38 to 1.92	< .001
Partial epilepsy (vs generalized), trial mean^b^	Trial‐level covariate	1.47	0.99 to 2.19	0.06
Partial epilepsy (vs generalized), centered^a^ * VP (vs CBZ)	Intervention‐covariate interaction	1.36	0.97 to 1.89	0.07
				

VP: Valproate, CBZ: Carbamazepine, HR: Hazard ratio, given by *exp*(*β*), CI: Confidence interval. Standard deviations of random intercept (ie, frailty) and random effect of VP (vs CBZ) equal 0.126 and 0.164, respectively. *P*‐values are for Wald type tests of the null hypothesis that the log HR equals zero.

a Covariates are centered within trials, to avoid ecological bias (see 88).

b Trial mean value for the covariate is entered in the analysis, to quantify the bias that would occur if centering of the covariate were not performed.

A recent investigation of the intervention‐covariate interaction on the time to remission of epilepsy demonstrated that bias occurs when within‐trial and across‐trial information is not separated. 88 Such separation can be performed by centering the covariates, hence we have centered the covariates in in our analysis (Table 8). The possible bias that may occur when within‐trial and across‐trial information are amalgamated can be quantified by including the trial‐mean in the model, 88 as we have done here (Table 8).

## 7 DISCUSSION

Our search has identified a wide range of articles on topics regarding TTE IPD‐MA, and is the first comprehensive review on this topic to our knowledge. However, the basics of the methodology regarding TTE data was excluded from our search as it did not concern MA or clustered data. Covering all methodological works regarding TTE data would have been an immense task. As such, we were forced to include relevant literature based on our own opinion to introduce this topic, and restrict our systematic search through Pubmed and Web of Science to works that simultaneously concerned IPD, meta‐analysis and time‐to‐event data. We did not cover every article that covers these three topics, as this was not our aim. Instead, we our purpose was to achieve theoretical saturation, that is, that an extended search would be unlikely to add important information.

The general consensus in the reviewed works was that the Cox model should be the default model of choice for TTE IPD‐MA. Though, it is also criticized for not yielding a valid estimate of intervention effect when not all (un‐)measured predictive covariates are accounted for, mostly on theoretical grounds. The literature is currently missing information on the impact of this issue in real life data, leading us to suggest that further research should focus thereon. As such, we have provided a comprehensive review of current methods for IPD‐MA of TTE data.

Although the statistical properties of the meta‐analysis estimators for the two‐stage approach have been well studied and simulation studies have investigated the performance for meta‐analysis of dichotomous and continuous outcome data, this is not the case for time‐to‐event data. Further, although aggregate data (ie, estimates from the literature) can readily be included in the two‐stage approach (provided that the models are specified the same), as well as in Bayesian one‐stage models, there appears to be no method yet for doing so in a Frequentist model.

Another issue is to what extend one should try to borrow information across trials in the one‐approach. In the two‐stage approach, no information is borrowed (apart from the intervention effect and its uncertainty), as all parameters are naturally estimated per trial. To what extend one should account for this in the one‐stage approach, by stratifying the baseline and covariate effects or by applying random effects and a frailty, deserves extra attention in the literature. For the meta‐analysis of trials with adequate sample sizes, the safest choice is to stratify all included parameters as this accounts for all differences in baselines between trials. In a simulation study where IPD from a total of 600 participants from 3‐20 trials were generated, both the frailty and the stratified baseline method worked well, 29 though exactly what sample sizes are necessary for this strategy, and especially for the stratification of covariates as well, has apparently not yet been identified.

## 8 CONCLUDING REMARKS

We have discussed numerous models in this manuscript, the choice between which is not always straightforward. For this reason, we provide some recommendations below. First, intervention effect conditional on covariates and/or frailties have different interpretations from marginal ones (ie, averaged over the entire sample and follow‐up time), and yield different estimates. Before embarking on an IPD‐MA, researchers should decide whether a conditional or a marginal effect is of interest. As assumptions may be satisfied on one scale but not the other, this may lead to a different choice of model.

Additionally, one can choose between one‐stage and two‐stage models. In the two‐stage method participants within trials are compared, which inherently yields a conditional intervention effect and stratified baselines. The one‐stage approach offers more possibilities as it allows for conditional intervention effects as well as marginal ones, and frailties for the baseline. When the same (or similar) model assumptions are made for these models and the same estimation methods are used, these two approaches generally lead to the same estimates of intervention effect. 13, 28 Though, the one‐stage approach can have better convergence properties when the included studies are very small, 28, 150 or at least one of the studies has zero events.

Further, when a conditional effect is desired (in contrast to a marginal one), we recommend to apply random effects instead of common effects, as common effects models are only valid when no heterogeneity is present, which is unlikely in our experience. When a marginal effect is desired, only a correction for the variance is necessary. As described in section 2, when an intervention effect is present the estimated intervention effect in PH models may be time‐dependent, depending on the distribution of prognostic factors that are not accounted for (even if balanced across intervention groups). This may lead to heterogeneity in intervention effects across trials that have different follow‐up lengths. Further, differences in trial design and methodology and clinical procedures may contribute to the heterogeneity of the intervention effect. 12 Random effects models can account for heterogeneity of the intervention effect and lead to the same solution as common effect models when no heterogeneity is present. However, if a formal test of heterogeneity is desired, a variety of tests can be used. For one‐stage meta‐analysis, the common effect model (without trial effects) is nested in the frailty model, and therefore a comparison of these models can be made using the log‐likelihood ratio test. 14 Alternatively, a score test, 151, 152, 153 or a small sample test can be used. 154 A permutation test for testing of the presence of heterogeneity in time‐to‐event data was recently proposed, and a simulation showed that the method is more powerful and has a better type I error rate than likelihood ratio tests of a random effect. 155

Finally, when comparing non‐nested (eg, PH vs AFT) models, more general methods are needed. In such cases, one may select the model with lowest value for Akaike's Information Criterion (AIC) 156, 157 or the Bayesian Information Criterion (BIC) 156, 157, 158. Though, due to the correlated nature of participants within trials a correction for clustering should be made, which is not straightforward in the frequentist estimation framework as quantification of the number of degrees of freedom is difficult. For subject‐specific inferences, the conditional (cAIC) can be used, whereas for inferences on the population level the marginal AIC can be used. 64, 74, 159, 160

Further, one should be cautious regarding model selection. If one model is rejected, bias will appear in the estimated intervention effect and significance in a second model if the second model is not independent of the test that was used to reject the first, such as when a non‐PH effect is included in the model after a statistical test indicated non‐proportionality. 161 This bias can be alleviated by bootstrapping the model selection procedure. On the other hand, this bias does not occur when the second model is independent of the test used to reject the first model. 161

## CONFLICT OF INTEREST

The author reported no conflict of interest.

## Supporting information


**Appendix** **S1**: Supporting Information

## Data Availability

The data that support the findings of this study are not publicly available, according to the conditions determined by the Epilepsy Monotherapy Trial Group, but are available on request from AM, by e‐mailing A.G.Marson@liverpool.ac.uk.
