# Supplementary material for: Individual participant data meta‐analysis of intervention studies with time‐to‐event outcomes: A review of the methodology and an applied example
Source: Res Synth Methods. 2020 Feb 6;11(2):148–68. doi: 10.1002/jrsm.1384 (PMC7079159; doi:10.1002/jrsm.1384)
Supplement: Supplementary file 1 — Appendix S1: Supporting Information [file JRSM-11-148-s001.pdf]

Supporting Information for Individual participant data meta-analysis of intervention studies with time-to-event outcomes: A review of the methodology and an applied example

Valentijn M.T. de Jong, Karel G.M. Moons, Richard D. Riley, Catrin Tudur Smith, Anthony G. Marson, Marinus J.C. Eijkemans, Thomas P.A. Debray

July 12, 2019

1 Search filters

1.1 Search filter for Pubmed

(”individual patient” OR ”individual participant” OR ”IPD” OR ”patient level data” OR “patient-level data”)
AND
(”meta-analysis” OR ”meta-analyses” OR ”systematic review” OR hierarchical OR cluster OR ”multi-level” OR ”mixed model” OR ”mixed effect” OR ”mixed effects” OR ”random coefficient” OR ”random-effects model” OR ”random-parameter model”)
AND
(”survival” OR Cox OR ”Royston-Parmar” OR Weibull OR Failure OR Hazard OR hazards OR AFT OR Frailty OR ”time-to-event” OR ”Kaplan-Meier” OR Gompertz)

1.2 Search Filter for advanced search of Web of Science

(TS=((”individual patient” OR ”individual participant” OR ”IPD” OR ”patient level data” OR patient-level data)
AND
(”meta-analysis” OR ”meta-analyses” OR ”systematic review” OR hierarchical OR cluster OR ”multi-level” OR mixed model OR mixed effect OR mixed effects OR random coefficient OR random-effects model OR random-parameter model)
AND
(survival OR Cox OR Royston-Parmar OR Weibull OR Failure OR Hazard OR hazards OR AFT OR Frailty OR time-to-event OR Kaplan-Meier OR Gompertz)))

2 Code used in applied example

This code is also available from `https://github.com/VMTdeJong/Epilepsy`

2.1 The data

```
library(survminer)


# Kaplan meier
km <- survfit(Surv(SEZTIME, SCENS) ~  Drug + Epilepsy, data = epi)
ggsurvplot(km)

# Some new functions
# Center a variable
Center <- function(x, trial.id) {
  for (trial in sort(unique(trial.id)))
  {
    selection.id <- trial.id == trial
    selection <- x[selection.id]
    x[selection.id] <- selection - mean(selection, na.rm = T)
  }
  x
}

# Trial mean of a variable
TrialMean  <- function(x, trial.id) {
  for (trial in sort(unique(trial.id)))
  {
    selection.id <- trial.id == trial
    x[selection.id] <- mean(x[selection.id], na.rm = T)
  }
  x
}

# Computing the centered and trial mean variables
epi$EPTYPE.trialmean  <- TrialMean(epi$EPTYPE,  epi$TRIAL)
epi$EPTYPE.center     <- Center(epi$EPTYPE,  epi$TRIAL)
```

2.2 Model fitting

```
# Some new functions
# Extract the standard error from a model fit.
se <- function(object)
  sqrt(diag(vcov(object)))

# Extract the confidence interval from a coxme fit.
confint.coxme <- function(object, level = .95, digits = 2) {
  z <- qnorm(1 - (1 - level)/2)
  b <- coef(object)
  s <- se(object)

  ci.lb <- b - z * s
  ci.ub <- b + z * s

  out <- data.frame(b, ci.lb, ci.ub, s, exp(b), exp(ci.lb), exp(ci.ub))
```

```
  out <- round(out, digits = digits)
  colnames(out) <- c("coef", "ci.lb(coef)", "ci.ub(coef)", "se(coef)",
                     "exp(coef)", "ci.lb(exp(coef))", "ci.ub(exp(coef))")
  out$`Wald p` <- round(pnorm(b/s, lower.tail = F) * 2, digits + 1)

  out$CI <- paste(out$`ci.lb(exp(coef))`, " to ", out$`ci.ub(exp(coef))`,
                  sep = "", collapse = NULL)
  out
}

# Running the Cox models
library(coxme)
cox.drug.ri.re <- coxme(Surv(SEZTIME, SCENS) ~ DRUG + (1 + DRUG |TRIAL),
                        data = epi)
cox.drug.ri.re.cov <- coxme(Surv(SEZTIME, SCENS)
                            ~ DRUG
                            + (1 + DRUG |TRIAL)
                            + EPTYPE.center
                            + EPTYPE.trialmean
                            + EPTYPE.center : DRUG
                            , data = epi)

# Applying our functions to obtain confidence intervals for the models
confint(cox.drug.ri.re)
confint(cox.drug.ri.re.cov)
```

2.3 Heterogeneity

The function MHRnormal is adapted from Austin PC, Wagner P, Merlo J. The median hazard ratio: a useful measure of variance and general contextual effects in multilevel survival analysis. Stat Med. November 2016. doi:10.1002/sim.7188

```
# Let var.re denote the estimate variance of the random effects,
# following a normal distribution.
MHRnormal <- function(var.re) exp(sqrt(var.re) * qnorm(0.75) * sqrt(2))

# Computing the MHR
MHRnormal(cox.drug.ri.re$vcoef[["TRIAL"]]["DRUG","DRUG"])
```

3 Overview of included articles

[1] Goldstein H, Browne W, Rasbash J. Multilevel modelling of medical data. *Statistics in Medicine.* 2002;21:3291–3315.

[2] Burke DL, Ensor J, Riley RD. Meta-analysis using individual participant data: one-stage and two-stage approaches, and why they may differ. *Statistics in Medicine.* 2017;36:855–875.

[3] Efthimiou O, Debray TP, Valkenhoef G, et al. GetReal in network meta-analysis: a review of the methodology. *Research Synthesis Methods.* 2016;7:236–263.

[4] Yau KKW, McGilchrist CA. ML and REML estimation in survival analysis with time dependent correlated frailty. *Statistics in Medicine.* 1998;17:1201–1213.

[5] Berry SM, Ishak KJ, Luce BR, Berry DA. Bayesian meta-analyses for comparative effectiveness and informing coverage decisions. *Medical Care.* 2010;48:S137–S144.

[6] Clayton DG. A Monte Carlo method for Bayesian inference in frailty models. *Biometrics.* 1991:467–485.

[7] Lanke J. How to Describe the Impact of the Family-Specific Frailty (Appendix of "Quantifying the Family Frailty Effect in Infant and Child Mortality by Using Median Hazard Ratio (MHR)"). *Historical Methods: A Journal of Quantitative and Interdisciplinary History.* 2010.

[8] Hougaard P. Modelling Heterogeneity in Survival Data. *Journal of Applied Probability.* 1991;28:695–701.

[9] Abbring JH, Berg VD, J G. The unobserved heterogeneity distribution in duration analysis. *Biometrika.* 2007;94:87–99.

[10] Bengtsson T, Dribe M. Quantifying the Family Frailty Effect in Infant and Child Mortality by Using Median Hazard Ratio (MHR). *Historical Methods: A Journal of Quantitative and Interdisciplinary History.* 2010;43:15–27.

[11] Vaupel JW, Manton KG, Stallard E. The Impact of Heterogeneity in Individual Frailty on the Dynamics of Mortality. *Demography.* 1979;16:439–454.

[12] Lambert P, Collett D, Kimber A, Johnson R. Parametric accelerated failure time models with random effects and an application to kidney transplant survival. *Statistics in Medicine.* 2004;23:3177–3192.

[13] Lin DY. Cox regression analysis of multivariate failure time data: the marginal approach. *Statistics in Medicine.* 1994;13:2233–2247.

[14] Spiekerman CF, Lin DY. Marginal regression models for multivariate failure time data. *Journal of the American Statistical Association.* 1998;93:1164–1175.

[15] Cai J, Prentice RL. Estimating Equations for Hazard Ratio Parameters Based on Correlated Failure Time Data. *Biometrika.* 1995;82:151–164.

[16] Claeskens G, Nguti R, Janssen P. One-sided tests in shared frailty models. *Test.* 2008;17:69–82.

[17] Economou P, Stehlík M. On Small Samples Testing for Frailty Through Homogeneity Test. *Communications in Statistics - Simulation and Computation.* 2015;44:40–65.

[18] Gray RJ. Tests for Variation Over Groups in Survival Data. *Journal of the American Statistical Association.* 1995;90:198–203.

[19] Shi Q, Sargent DJ. Meta-analysis for the evaluation of surrogate endpoints in cancer clinical trials. *International Journal of Clinical Oncology.* 2009;14:102–111.

[20] Barrett JK, Farewell VT, Siannis F, Tierney J, Higgins JPT. Two-stage meta-analysis of survival data from individual participants using percentile ratios. *Statistics in Medicine.* 2012;31:4296–4308.

[21] Marcucci M, Smith CT, Douketis JD, et al. Patient-level compared with study-level meta-analyses demonstrate consistency of D-dimer as predictor of venous thromboembolic recurrences. *Journal of Clinical Epidemiology.* 2013;66:415–425.

[22] Lin DY, Zeng D. On the relative efficiency of using summary statistics versus individual-level data in meta-analysis. *Biometrika.* 2010;97:321–332.

[23] Saramago P, Chuang LH, Soares MO. Network meta-analysis of (individual patient) time to event data alongside (aggregate) count data. *BMC Medical Research Methodology.* 2014;14:105.

[24] Resche-Rigon M, White IR, Bartlett JW, Peters SAE, Thompson SG, PROG-IMT Study Group . Multiple imputation for handling systematically missing confounders in meta-analysis of individual participant data. *Statistics in Medicine.* 2013;32:4890–4905.

[25] Crowther MJ, Look MP, Riley RD. Multilevel mixed effects parametric survival models using adaptive Gauss-Hermite quadrature with application to recurrent events and individual participant data meta-analysis. *Statistics in Medicine.* 2014;33:3844–3858.

[26] Hobbs BP, Sargent DJ, Carlin BP. Commensurate Priors for Incorporating Historical Information in Clinical Trials Using General and Generalized Linear Models. *Bayesian Analysis.* 2012;7:639–674.

[27] Renfro LA, Shi Q, Xue Y, Li J, Shang H, Sargent DJ. Center-Within-Trial Versus Trial-Level Evaluation of Surrogate Endpoints. *Computational Statistics & Data Analysis.* 2014;78:1–20.

[28] Lueza B, Rotolo F, Bonastre J, Pignon JP, Michiels S. Bias and precision of methods for estimating the difference in restricted mean survival time from an individual patient data meta-analysis. *BMC Medical Research Methodology.* 2016;16:37.

[29] Rondeau V, Michiels S, Liquet B, Pignon JP. Investigating trial and treatment heterogeneity in an individual patient data meta-analysis of survival data by means of the penalized maximum likelihood approach. *Statistics in Medicine.* 2008;27:1894–1910.

[30] Katsahian S, Latouche A, Mary JY, Chevret S, Porcher R. Practical methodology of meta-analysis of individual patient data using a survival outcome. *Contemporary Clinical Trials.* 2008;29:220–230.

[31] Riley RD, Thompson JR, Abrams KR. An alternative model for bivariate random-effects meta-analysis when the within-study correlations are unknown. *Biostatistics.* 2008;9:172–186.

[32] Riley RD, Price MJ, Jackson D, et al. Multivariate meta-analysis using individual participant data. *Research Synthesis Methods.* 2015;6:157–174.

[33] Debray TPA, Moons KGM, Valkenhoef G, et al. Get real in individual participant data (IPD) meta-analysis: a review of the methodology. *Research Synthesis Methods.* 2015;6:293–309.

[34] Wei Y, Royston P, Tierney JF, Parmar MKB. Meta-analysis of time-to-event outcomes from randomized trials using restricted mean survival time: application to individual participant data. *Statistics in Medicine.* 2015;34:2881–2898.

[35] Simmonds MC, Higgins JP, Stewart LA. Random-effects meta-analysis of time-to-event data using the expectation-maximisation algorithm and shrinkage estimators. *Research Synthesis Methods.* 2013;4:144–155.

[36] Bowden J, Tierney JF, Simmonds M, Copas AJ, Higgins JP. Individual patient data meta-analysis of time-to-event outcomes: one-stage versus two-stage approaches for estimating the hazard ratio under a random effects model. *Research Synthesis Methods.* 2011;2:150–162.

[37] Simmonds MC, Tierney J, Bowden J, Higgins JP. Meta-analysis of time-to-event data: a comparison of two-stage methods. *Research Synthesis Methods.* 2011;2:139–149.

[38] Buyse M, Molenberghs G, Paoletti X, et al. Statistical evaluation of surrogate endpoints with examples from cancer clinical trials. *Biometrical Journal.* 2016;58:104–132.

[39] Lueza B, Mauguen A, Pignon JP, Rivero-Arias O, Bonastre J, MAR-LC Collaborative Group . Difference in Restricted Mean Survival Time for Cost-Effectiveness Analysis Using Individual Patient Data Meta-Analysis: Evidence from a Case Study. *PloS One.* 2016;11:e0150032.

[40] Lueza B, Rotolo F, Bonastre J, Pignon JP, Michiels S. Erratum to: Bias and precision of methods for estimating the difference in restricted mean survival time from an individual patient data meta-analysis. *BMC Medical Research Methodology.* 2016;16:71.

[41] Sobel M, Madigan D, Wang W. Causal Inference for Meta-Analysis and Multi-Level Data Structures, with Application to Randomized Studies of Vioxx. *Psychometrika.* 2016.

[42] Veroniki AA, Straus SE, Soobiah C, Elliott MJ, Tricco AC. A scoping review of indirect comparison methods and applications using individual patient data. *BMC Medical Research Methodology.* 2016;16:47.

[43] Song F, Bachmann MO. Cumulative subgroup analysis to reduce waste in clinical research for individualised medicine. *BMC medicine.* 2016;14:197.

[44] Moore RA, Edwards JE, McQuay HJ. Acute pain: individual patient meta-analysis shows the impact of different ways of analysing and presenting results. *Pain.* 2005;116:322–331.

[45] Lyman GH, Kuderer NM. The strengths and limitations of meta-analyses based on aggregate data. *BMC Medical Research Methodology.* 2005;5:14.

[46] Michiels S, Baujat B, Mahé C, Sargent DJ, Pignon JP. Random effects survival models gave a better understanding of heterogeneity in individual patient data meta-analyses. *Journal of Clinical Epidemiology.* 2005;58:238–245.

[47] Tierney JF, Stewart LA. Investigating patient exclusion bias in meta-analysis. *International Journal of Epidemiology.* 2005;34:79–87.

[48] Berlin JA, Santanna J, Schmid CH, Szczech LA, Feldman HI. Individual patient- versus group-level data meta-regressions for the investigation of treatment effect modifiers: ecological bias rears its ugly head. *Statistics in Medicine.* 2002;21:371–387.

[49] Duchateau L, Pignon JP, Bijnens L, Bertin S, Bourhis J, Sylvester R. Individual Patient- versus Literature-Based Meta-analysis of Survival Data: Time to Event and Event Rate at a Particular Time Can Make a Difference, an Example Based on Head and Neck Cancer. *Controlled Clinical Trials.* 2001;22:538–547.

[50] Trikalinos TA, Ioannidis JPA. Predictive modeling and heterogeneity of baseline risk in meta-analysis of individual patient data. *Journal of Clinical Epidemiology.* 2001;54:245–252.

[51] Johnson B, Carlin BP, Hodges JS. Cross-Study Hierarchical Modeling of Stratified Clinical Trial Data. *Journal of Biopharmaceutical Statistics.* 1999;9:617–640.

[52] Donohue MC, Overholser R, Xu R, Vaida F. Conditional Akaike information under generalized linear and proportional hazards mixed models. *Biometrika.* 2011;98:685–700.

[53] DerSimonian R, Laird N. Meta-analysis in clinical trials. *Controlled Clinical Trials.* 1986;7:177–188.

[54] Stewart LA, Parmar MKB. Meta-analysis of the literature or of individual patient data: is there a difference?. *The Lancet.* 1993;341:418–422.

[55] Riley RD, Simmonds MC, Look MP. Evidence synthesis combining individual patient data and aggregate data: a systematic review identified current practice and possible methods. *Journal of Clinical Epidemiology.* 2007;60:431.e1–431.e12.

[56] Riley RD, Lambert PC, Abo-Zaid G. Meta-analysis of individual participant data: rationale, conduct, and reporting. *Bmj.* 2010;340:c221.

[57] Aalen O. Effects of frailty in survival analysis. *Statistical Methods in Medical Research.* 1994;3:227–243.

[58] Keiding N, Andersen PK, Klein JP. The Role of Frailty Models and Accelerated Failure Time Models in Describing Heterogeneity Due to Omitted Covariates. *Statistics in Medicine.* 1997;16:215–224.

[59] Vonesh EF, Greene T, Schluchter MD. Shared parameter models for the joint analysis of longitudinal data and event times. *Statistics in Medicine.* 2006;25:143–163.

[60] Munda M, Legrand C. Adjusting for centre heterogeneity in multicentre clinical trials with a time-to-event outcome. *Pharmaceutical Statistics.* 2014;13:145–152.

[61] Commenges D, Andersen PK. Score test of homogeneity for survival data. *Lifetime Data Analysis.* 1995;1:145–156; discussion 157–159.

[62] Sargent DJ. A general framework for random effects survival analysis in the Cox proportional hazards setting. *Biometrics.* 1998;54:1486–1497.

[63] Rondeau V, Filleul L, Joly P. Nested frailty models using maximum penalized likelihood estimation. *Statistics in Medicine.* 2006;25:4036–4052.

[64] Legrand C, Ducrocq V, Janssen P, Sylvester R, Duchateau L. A Bayesian approach to jointly estimate centre and treatment by centre heterogeneity in a proportional hazards model. *Statistics in Medicine.* 2005;24:3789–3804.

[65] Glidden DV, Vittinghoff E. Modelling clustered survival data from multicentre clinical trials. *Statistics in Medicine.* 2004;23:369–388.

[66] Bennett MM, Crowe BJ, Price KL, Stamey JD, Seaman JW. Comparison of Bayesian and frequentist meta-analytical approaches for analyzing time to event data. *Journal of Biopharmaceutical Statistics.* 2013;23:129–145.

[67] Biard L, Porcher R, Resche-Rigon M. Permutation tests for centre effect on survival endpoints with application in an acute myeloid leukaemia multicentre study. *Statistics in Medicine.* 2014;33:3047–3057.

[68] Austin PC, Wagner P, Merlo J. The median hazard ratio: a useful measure of variance and general contextual effects in multilevel survival analysis. *Statistics in Medicine.* 2016.

[69] Siannis F, Barrett JK, Farewell VT, Tierney JF. One-stage parametric meta-analysis of time-to-event outcomes. *Statistics in Medicine.* 2010;29:3030–3045.

[70] Crowther MJ, Riley RD, Staessen JA, Wang J, Gueyffier F, Lambert PC. Individual patient data meta-analysis of survival data using Poisson regression models. *BMC Medical Research Methodology.* 2012;12:34.

[71] Renfro LA, Shi Q, Sargent DJ, Carlin BP. Bayesian adjusted R2 for the meta-analytic evaluation of surrogate time-to-event endpoints in clinical trials. *Statistics in Medicine.* 2012;31:743–761.

[72] Thompson S, Kaptoge S, White I, et al. Statistical methods for the time-to-event analysis of individual participant data from multiple epidemiological studies. *International Journal of Epidemiology.* 2010;39:1345–1359.

[73] Poppe KK, Doughty RN, Yu CM, et al. Understanding differences in results from literature-based and individual patient meta-analyses: An example from meta-analyses of observational data. *International Journal of Cardiology.* 2011;148:209–213.

[74] Boutitie F, Gueyffier F, Pocock SJ, Boissel JP. Assessing treatment–time interaction in clinical trials with time to event data: a meta-analysis of hypertension trials. *Statistics in Medicine.* 1998;17:2883–2903.

[75] Messori A. Current controversies in the application of meta—analysis, (with special reference to oncological treatments). *Pharmacy World and Science.* 1997;19:152–158.

[76] Collaboration TFS. Systematically missing confounders in individual participant data meta-analysis of observational cohort studies. *Statistics in Medicine.* 2009;28:1218.

[77] Shi Q, Renfro LA, Bot BM, Burzykowski T, Buyse M, Sargent DJ. Comparative assessment of trial-level surrogacy measures for candidate time-to-event surrogate endpoints in clinical trials. *Computational Statistics & Data Analysis.* 2011;55:2748–2757.

[78] Fisher DJ, others . Two-stage individual participant data meta-analysis and generalized forest plots. *Stata Journal.* 2015;15:369–396.

[79] Huang CY, Qin J, Tsai HT. Efficient Estimation of the Cox Model With Auxiliary Subgroup Survival Information. *Journal of the American Statistical Association.* 2015:00–00.

[80] Freeman SC, Fisher D, Tierney JF, Carpenter JR. A framework for identifying treatment-covariate interactions in individual participant data network meta-analysis. *Research Synthesis Methods.* 2018.

[81] Saramago P, Sutton AJ, Cooper NJ, Manca A. Mixed treatment comparisons using aggregate and individual participant level data. *Statistics in Medicine.* 2012;31:3516–3536.

[82] Freeman SC, Carpenter JR. Bayesian one-step IPD network meta-analysis of time-to-event data using Royston-Parmar models. *Research Synthesis Methods.* 2017;8:451–464.

[83] Higgins JP, Thompson SG, Spiegelhalter DJ. A re-evaluation of random-effects meta-analysis. *Journal of the Royal Statistical Society: Series A (Statistics in Society).* 2009;172:137–159.

[84] Riley RD, Higgins JPT, Deeks JJ. Interpretation of random effects meta-analyses. *BMJ.* 2011;342:d549.

[85] Veroniki AA, Jackson D, Viechtbauer W, et al. Methods to estimate the between-study variance and its uncertainty in meta-analysis. *Research Synthesis Methods.* 2016;7:55–79.

[86] Paule RC, Mandel J. Consensus values and weighting factors. *Journal of Research of the National Bureau of Standards.* 1982;87:377–385.

[87] DerSimonian R, Kacker R. Random-effects model for meta-analysis of clinical trials: an update. *Contemporary Clinical Trials.* 2007;28:105–114.

[88] Bowden J, Tierney JF, Copas AJ, Burdett S. Quantifying, displaying and accounting for heterogeneity in the meta-analysis of RCTs using standard and generalised Q statistics. *BMC Medical Research Methodology.* 2011;11:41.

[89] Viechtbauer W. Confidence intervals for the amount of heterogeneity in meta-analysis. *Statistics in Medicine.* 2007;26:37–52.

[90] Sidik K, Jonkman JN. A comparison of heterogeneity variance estimators in combining results of studies. *Statistics in Medicine.* 2007;26:1964–1981.

[91] Knapp G, Hartung J. Improved tests for a random effects meta-regression with a single covariate. *Statistics in Medicine.* 2003;22:2693–2710.

[92] Brockwell SE, Gordon IR. A comparison of statistical methods for meta-analysis. *Statistics in Medicine.* 2001;20:825–840.

[93] Normand SLT. Meta-analysis: formulating, evaluating, combining, and reporting. *Statistics in Medicine.* 1999;18:321–359.

[94] Sidik K, Jonkman JN. Robust variance estimation for random effects meta-analysis. *Computational Statistics & Data Analysis.* 2006;50:3681–3701.

[95] Hartung J. An alternative method for meta-analysis. *Biometrical Journal.* 1999;41:901–916.

[96] IntHout J, Ioannidis JP, Borm GF. The Hartung-Knapp-Sidik-Jonkman method for random effects meta-analysis is straightforward and considerably outperforms the standard DerSimonian-Laird method. *BMC Medical Research Methodology.* 2014;14:25.

[97] Cornell JE, Mulrow CD, Localio R, et al. Random-effects meta-analysis of inconsistent effects: a time for change. *Annals of Internal Medicine.* 2014;160:267–270.

[98] McGilchrist CA. REML estimation for survival models with frailty. *Biometrics.* 1993:221–225.

[99] Ripatti S, Palmgren J. Estimation of multivariate frailty models using penalized partial likelihood. *Biometrics.* 2000;56:1016–1022.

[100] Therneau TM, Grambsch PM, Pankratz VS. Penalized survival models and frailty. *Journal of computational and graphical statistics.* 2003;12:156–175.

[101] Debray TPA, Moons KGM, Ahmed I, Koffijberg H, Riley RD. A framework for developing, implementing, and evaluating clinical prediction models in an individual participant data meta-analysis. *Statistics in Medicine.* 2013;32:3158–3180.

[102] Riley RD, Jackson D, Salanti G, et al. Multivariate and network meta-analysis of multiple outcomes and multiple treatments: rationale, concepts, and examples. *BMJ.* 2017;358:j3932.

[103] Morris C, Christiansen C. Fitting Weibull duration models with random effects. *Lifetime Data Analysis.* 1995;1:347–359.

[104] Vaida F, Xu R. Proportional hazards model with random effects. *Statistics in Medicine.* 2000;19:3309–3324.

[105] Hartung J, Knapp G. On confidence intervals for the among-group variance in the one-way random effects model with unequal error variances. *Journal of Statistical Planning and Inference.* 2005;127:157–177.

[106] Langan D, Higgins JPT, Jackson D, et al. A comparison of heterogeneity variance estimators in simulated random-effects meta-analyses. *Research Synthesis Methods.* 2018.

[107] Jackson D, Law M, Rücker G, Schwarzer G. The Hartung-Knapp modification for random-effects meta-analysis: A useful refinement but are there any residual concerns?. *Statistics in Medicine.* 2017;36:3923–3934.

[108] Hardy R, Thompson SG. A likelihood approach to meta-analysis with random effects. *Statistics in Medicine.* 1996;15:619–629.

[109] Sudell M, Tudur Smith C, Gueyffier F, Kolamunnage-Dona R. Investigation of 2-stage meta-analysis methods for joint longitudinal and time-to-event data through simulation and real data application. *Statistics in Medicine.* 2018;37:1227–1244.

[110] Buyse M. Use of Meta-Analysis for the Validation of Surrogate Endpoints and Biomarkers in Cancer Trials:. *The Cancer Journal.* 2009;15:421–425.

[111] Viechtbauer W. Conducting meta-analyses in R with the metafor package. *Journal of Statistical Software.* 2010;36.

[112] Thompson SG, Sharp SJ. Explaining heterogeneity in meta-analysis: a comparison of methods. *Statistics in Medicine.* 1999;18:2693–2708.

[113] Kelley GA, Kelley KS. Statistical models for meta-analysis: A brief tutorial. *World Journal of Methodology.* 2012;2:27.

[114] Tudur Smith C, Williamson PR. A comparison of methods for fixed effects meta-analysis of individual patient data with time to event outcomes. *Clinical Trials.* 2007;4:621–630.

[115] Tudur Smith C, Williamson PR, Marson AG. An overview of methods and empirical comparison of aggregate data and individual patient data results for investigating heterogeneity in meta-analysis of time-to-event outcomes. *Journal of Evaluation in Clinical Practice.* 2005;11:468–478.

[116] Tudur Smith C, Williamson PR, Marson AG. Investigating heterogeneity in an individual patient data meta-analysis of time to event outcomes. *Statistics in Medicine.* 2005;24:1307–1319.

[117] Brilleman SL, Crowther MJ, Moreno-Betancur M, et al. Joint longitudinal and time-to-event models for multilevel hierarchical data. *Statistical Methods in Medical Research.* 2018;0:1–14.

[118] Jung TH, Peduzzi P, Allore H, Kyriakides TC, Esserman D. A joint model for recurrent events and a semi-competing risk in the presence of multi-level clustering. *Statistical Methods in Medical Research.* 2018;0:1–15.

[119] Localio AR, Berlin JA, Ten Have TR, Kimmel SE. Adjustments for center in multicenter studies: an overview. *Annals of Internal Medicine.* 2001;135:112–123.

[120] Rotolo F, Paoletti X, Michiels S. surrosurv: An R package for the evaluation of failure time surrogate endpoints in individual patient data meta-analyses of randomized clinical trials. *Computer Methods and Programs in Biomedicine.* 2018;155:189–198.

[121] Paxton EW, Mohaddes M, Laaksonen I, et al. Meta-analysis of individual registry results enhances international registry collaboration. *Acta Orthopaedica.* 2018;89:369–373.

[122] Tierney JF, Vale C, Riley R, et al. Individual participant data (IPD) meta-analyses of randomised controlled trials: guidance on their use. *PLoS Medicine.* 2015;12:e1001855.

[123] Hua H, Burke DL, Crowther MJ, Ensor J, Tudur Smith C, Riley RD. One-stage individual participant data meta-analysis models: estimation of treatment-covariate interactions must avoid ecological bias by separating out within-trial and across-trial information. *Statistics in Medicine.* 2017;36:772–789.

[124] Langan D, Higgins JPT, Simmonds M. Comparative performance of heterogeneity variance estimators in meta-analysis: a review of simulation studies: A Review of Simulation Studies. *Research Synthesis Methods.* 2017;8:181–198.

[125] Veroniki AA, Jackson D, Bender R, et al. Methods to calculate uncertainty in the estimated overall effect size from a random-effects meta-analysis. *Research Synthesis Methods.* 2019;10:23–43.

[126] Sidik K, Jonkman JN. A simple confidence interval for meta-analysis. *Statistics in Medicine.* 2002;21:3153–3159.

[127] Lee KH, Dominici F, Schrag D, Haneuse S. Hierarchical models for semicompeting risks data with application to quality of end-of-life care for pancreatic cancer. *Journal of the American Statistical Association.* 2016;111:1075–1095.

[128] Schnell P, Bandyopadhyay D, Reich BJ, Nunn M. A marginal cure rate proportional hazards model for spatial survival data. *Journal of the Royal Statistical Society: Series C (Applied Statistics).* 2015;64:673–691.
